# Editorial: Digital medicine in psychiatry and neurology—from mobile monitoring to scalable, equitable care

**DOI:** 10.3389/fpsyt.2026.1944482

**Published:** 2026-08-18

**Authors:** David M. A. Mehler, Simon H. Kohl, Lars Masanneck

**Affiliations:** 1 Department of Psychiatry, Psychotherapy and Psychosomatics, Faculty of Medicine, RWTH Aachen University, Aachen, Germany; 2 Institute for Translational Psychiatry, University of Münster, Münster, Germany; 3 Cardiff University Brain Research Imaging Centre (CUBRIC), School of Psychology, Cardiff University, Cardiff, United Kingdom; 4 Child Neuropsychology Section, Department of Child and Adolescent Psychiatry, Psychosomatics and Psychotherapy, Faculty of Medicine, RWTH Aachen University, Aachen, Germany; 5 Translational Neuroscience, Institute of Neuroscience and Medicine (INM-10), Forschungszentrum Jülich, Jülich, Germany; 6 Department of Neurology, University Hospital and Medical Faculty University Hospital Düsseldorf, Düsseldorf, Germany

**Keywords:** artificial intelligence (AI), child psychiatry, digital medicine, digital monitoring, neurology, psychiatry

Mental and neurological diseases represent a substantial and growing global health burden, yet care still depends heavily on episodic encounters that often fail to capture the dynamic fluctuations of symptoms, behaviors, and functional impairments that unfold in everyday life. Against this backdrop, digital medicine—including smartphones, wearable sensors, artificial intelligence (AI), virtual reality, and internet-based interventions—offers continuous monitoring, personalized treatment, and scalable delivery of care beyond traditional clinical settings.

The contributions in this Research Topic illustrate both the promise and complexity of this transformation. Across psychiatry and neurology, the included studies demonstrate how digital technologies can be used to measure clinically relevant phenomena in real-world environments, support prevention and treatment, and facilitate more personalized approaches to care. At the same time, they highlight important challenges related to implementation, engagement, digital literacy, accessibility, and long-term adoption.

Taken together, these contributions do more than showcase individual technologies. They trace a translational pathway for digital medicine: from monitoring and prediction, through digital interventions, to the human and systemic factors that decide whether innovation achieves clinical impact in routine care.

A central promise of digital medicine lies in its ability to capture clinically meaningful information continuously in ecologically valid settings. Mascia et al. evaluated whether behavioral observations and wearable sensors can predict severe emotional outbursts in children aged 4–8 years. Early rough behaviors predicted later outbursts, while wearable monitoring was well tolerated, demonstrating the potential of combining behavioral observations and wearable sensor data to identify early indicators of emotional dysregulation in pediatric populations.

Kuijpers et al. present a proof-of-concept study evaluating a smartphone-based eye-scanning application for detecting benzodiazepine use by measuring drug-induced impairments in eye convergence. Using individualized baseline measurements, the proposed approach achieved high classification performance, demonstrating the potential of personalized digital biomarkers for remote substance use monitoring.

The COMFORTage protocol by Valero et al. exemplifies the translational pathway from data capture to AI-enabled prevention. In this pilot study, multimodal clinical, biological, imaging, and speech data are combined with digital cognitive and functional stimulation for individuals with mild cognitive impairment or mild Alzheimer’s disease dementia, advancing scalable approaches to personalized monitoring, prediction, and intervention in neurodegenerative disorders. With emerging disease-modifying therapies, improved digital monitoring may facilitate earlier diagnosis and personalized treatment.

Together, these studies demonstrate how continuous, objective, and context-sensitive digital data may enable earlier detection of clinical deterioration, more precise phenotyping, and ultimately more personalized healthcare.

As digital technologies become increasingly capable of analyzing behavioral data and interacting directly with users, questions arise regarding their future role in mental healthcare. Sinha et al. conducted a mixed-methods retrospective observational study evaluating the AI-based mental health chatbot Wysa on Singapore’s national digital mental health platform during the COVID-19 pandemic. Among more than 69,000 website users and 4,100 app users, brief web engagement increased app use and retention, while most users successfully achieved cognitive restructuring. The findings support scalable AI-assisted mental health interventions to enhance engagement and broaden access to psychological support.

Virto-Farfan et al. conducted a randomized pilot trial evaluating Virtual Reality Stress Inoculation Training (VR-SIT) as an adjunct to standard treatment for mild-to-moderate major depressive disorder. Overall, 54 participants received either standard pharmacological and psychotherapeutic treatment alone or combined with VR-SIT. Both groups showed comparable reductions in depressive symptoms, but the VR-SIT group demonstrated significantly greater improvements in motivation and treatment engagement, supporting VR as a promising adjunct to conventional depression therapy.

Miró et al. presented the protocol for DigiDOL-Ad, a multi-phase project to develop and evaluate a digital psychosocial intervention for adolescents with chronic pain. Combining stakeholder co-creation, iterative usability testing, and multicenter evaluation, the protocol highlights the importance of involving multiple stakeholders throughout the development and implementation process.

Sanchez Roman et al. evaluated the usability of a CBT-based smartphone intervention for adolescents with anorexia nervosa or bulimia nervosa. Across healthy adolescents and patients with eating disorders, the app achieved high usability and acceptance. The findings demonstrate the importance of user-centered design and suggest that digital interventions may help bridge treatment gaps while awaiting outpatient psychotherapy.

Nacke et al. conducted a dissemination trial evaluating tailored online eating disorder prevention programs in 3,654 women from the general population. Interventions based on cognitive-behavioral principles significantly reduced weight concerns and eating disorder symptoms while improving intuitive eating, with some benefits maintained for up to 12 months. Despite moderate adherence, the study demonstrates the feasibility and scalability of risk-tailored, internet-based prevention programs for eating disorders.

A systematic review by Alsalloum et al. including 29 studies and clinical trials (>5,000 participants) examined personalized digital health interventions for depression. Machine learning models using mobile sensing and ecological momentary assessment improved mood prediction, while just-in-time adaptive interventions demonstrated moderate-to-large reductions in depression, anxiety, and repetitive negative thinking. The review concludes that personalized digital interventions can address depression heterogeneity and support more precise, scalable, and effective mental healthcare while highlighting implementation and equity challenges.

Lastly, the opinion article by Zhang and Wang examines whether advances in artificial intelligence could enable digital systems to perform functions traditionally associated with psychotherapists. The authors discuss the potential of AI to improve access, scalability, and personalization of mental healthcare while emphasizing challenges related to empathy, therapeutic alliance, algorithmic bias, privacy, accountability, and ethical governance, highlighting the need for careful integration of AI into clinical practice.

Together, these studies suggest that digital interventions are moving beyond digitization of existing therapies. They increasingly leverage the unique capabilities of digital platforms—including personalization, scalability, continuous adaptation, real-time responsiveness, and AI-informed interactive tools—to address the heterogeneity of psychiatric and neurological conditions.

Successful transformation of healthcare through technological innovation requires that individuals can access, understand, trust, adopt, and sustain engagement with digital tools. By examining digital health literacy in individuals with bipolar disorder, Kokwaro et al. highlight that access to digital mental health does not automatically translate into usable care. Their findings reveal a polarized literacy profile and show that structural and access-related factors, rather than symptom severity, shape digital engagement, highlighting the need for tailored implementation support.

A cross-sectional survey by Mazuz of 86 Israeli adults aimed to identify factors influencing adoption of the Hebrew PTSD Coach mobile app following the October 7, 2023, terrorist attack. Trauma literacy emerged as the strongest predictor of intention to use the app, while self-efficacy increased willingness to recommend it, highlighting the importance of psychoeducation and perceived capability for digital mental health adoption during emergencies.

Kaufmann et al. investigated reasons for non-participation and dropout in a longitudinal study of an app-based support service for psychiatric outpatients during the COVID-19 pandemic. Practical barriers, limited motivation, and illness-related factors were the main reasons for non-participation and attrition, identifying persistent barriers that continue to limit the effectiveness and generalizability of digital mental health interventions.

Viewed together, these contributions underscore a recurring theme across the field: implementation challenges are often human rather than technological. Factors such as literacy, motivation, perceived usefulness, trust, and symptom burden may ultimately determine whether digital innovations translate into real-world clinical benefit.

Collectively, the articles in this Research Topic map a field moving from digital measurement and prediction to personalized, scalable interventions, and ultimately toward the implementation challenges that determine real-world effectiveness. Advances in sensing technologies, mobile devices, and AI are enabling continuous collection of clinically meaningful data beyond traditional healthcare settings, while digital interventions are becoming increasingly personalized and adaptive across diverse psychiatric and neurological conditions. Successful implementation depends not only on technological performance but also on human factors including literacy, engagement, trust, and accessibility. At the same time, the evidence base remains uneven: proof-of-concept work is now abundant, but intervention studies need more rigorous controls to demonstrate specific effects and guide implementation.

Looking ahead, the central challenge is no longer whether digital technologies can contribute to mental and neurological healthcare, but how they can be integrated into routine care in ways that are effective, equitable, and sustainable. The contributions in this Research Topic provide important steps toward addressing this challenge and highlight promising directions for future research and implementation.

